# AhR-Mediated Effects of Dioxin on Neuronal Acetylcholinesterase Expression *in Vitro*

**DOI:** 10.1289/ehp.1206066

**Published:** 2013-02-20

**Authors:** Heidi Qunhui Xie, Hai-Ming Xu, Hua-Ling Fu, Qin Hu, Wen-Jing Tian, Xin-Hui Pei, Bin Zhao

**Affiliations:** State Key Laboratory of Environmental Chemistry and Ecotoxicology, Research Center for Eco-Environmental Sciences, Chinese Academy of Sciences, Beijing, China

**Keywords:** acetylcholinesterase (AChE), aryl hydrocarbon receptor (AhR), dioxin-responsive element (DRE), neuron, 2,3,7,8-tetrachlorodibenzo*-p-*dioxin (TCDD), transcriptional regulation

## Abstract

Background: Deficits in cognitive functioning have been reported in humans exposed to dioxins and dioxin-like compounds. Evidence suggests that dioxins induce cholinergic dysfunction mediated by hypothyroidism. However, little is known about direct effects of dioxins on the cholinergic system.

Objectives: We investigated the action of 2,3,7,8-tetrachlorodibenzo*-p-*dioxin (TCDD) on acetylcholinesterase (AChE), a key enzyme in cholinergic neurotransmission.

Methods: We used SK-N-SH human-derived neuronal cells to evaluate the effect of dioxin exposure on AChE.

Results: We consistently found a significant decrease in enzymatic activity of AChE in cultured neurons treated with TCDD. We also found that, unlike organophosphate pesticides that directly act on the catalytic center of AChE, the suppressive effect of dioxin was through transcriptional regulation. The addition of CH223191, an inhibitor of the aryl hydrocarbon receptor (AhR)-dependent pathway, counteracted the TCDD-induced suppression of AChE, suggesting involvement of the AhR-dependent pathway. The existence of putative dioxin-responsive element (DRE) consensus sequences in the human *ACHE* promoter region further supported this hypothesis. Consistent with the absence of DRE elements in mouse or rat *ACHE* promoter regions, suppression of AChE by TCDD did not occur in rat neuronal cells, indicating a potential species-specific effect.

Conclusions: In SK-N-SH cells, dioxin suppressed the activity of neuronal AChE via AhR-mediated transcriptional down-regulation. This is the first study to report direct interference by dioxin with the cholinergic neurotransmission system.

Polychlorinated dibenzo-*p*-dioxins (PCDDs), polychlorinated dibenzofurans (PCDFs), polychlorinated biphenyls (PCBs), and related dioxin-like compounds (DLCs) represent a diverse group of contaminants, many of which are highly toxic, and both environmentally and biologically persistent (reviewed by Mandal 2005). Dioxins and DLCs cause multiple toxic effects, including increased risk of cancer and interference with the function and development of the nervous, immune, and reproductive systems (Boucher et al. 2009; reviewed by Marinković et al. 2010, and by White and Birnbaum 2009).

Cholinergic neurotransmission and acetylcholinesterase (AChE; a vital functional enzyme in cholinergic neurotransmission) play important roles in multiple advanced brain functions, such as memory, learning, and attention (Hasselmo and Sarter 2011; reviewed by Soreq and Seidman 2001 and by Woolf and Butcher 2011). Emerging evidence suggests that maternal or perinatal exposure to dioxins or DLCs can interfere with the development of the central cholinergic system, including the development of AChE in the cerebellum (Ahmed 2011) and expression of muscarinic acetylcholine receptors in the cerebrum and cerebellum of rats (Coccini et al. 2007). Ahmed (2011) suggested that the effects of 2,3,7,8-tetrachlorodibenzo-*p*-dioxin (TCDD) on brain AChE were related to alterations in thyroid development. It is also plausible that dioxins could directly affect cholinergic neurotransmission, a possibility that we explored in the present study.

In general, activity of AChE can be affected in two ways: direct inhibition of enzymatic activity or suppression of transcription. Inhibition of AChE activity has been used as an indicator of organophosphorus insecticide (OP) exposure because OPs irreversibly inhibit the activity of AChE by binding to its catalytic residue (reviewed by Chen et al. 1999; Farahat et al. 2011). Therefore, we investigated the possibility that dioxin affects the enzymatic activity of AChE in cultured neurons, and further explored the possibility that this occurs by a transcriptional mechanism.

Dioxin is thought to exert its biological and toxicological effects primarily by binding to the aryl hydrocarbon receptor (AhR, dioxin receptor) followed by nuclear translocation and binding to dioxin-responsive elements (DREs) in gene promoters (reviewed by Beischlag et al. 2008). Putative DREs were observed in the promoter of the human *ACHE* gene but not in the mouse or rat *ACHE* genes (Sun et al. 2004), suggesting the possibility of species-specific effects of dioxins on AChE. Therefore, we also studied the role of the AhR-dependent pathway in dioxin-induced alterations of AChE and the species specificity of the effects.

## Materials and Methods

*Cell culture.* SK-N-SH cells (a cell line derived from human neuroblastoma cells) were purchased from the cell resource center of Chinese Academy of Medical Sciences (Beijing, China). SK-N-SH cells express both AChE and muscarinic acetylcholine receptor (Ezoulin et al. 2008; Pizzi et al. 2002; Popova and Rasenick 2004). Cells were maintained in Dulbecco’s modified Eagle’s medium (DMEM), supplemented with 10% fetal bovine serum (FBS), and incubated at 37°C in a water-saturated 5% CO_2_ incubator. PC12 cells [a cell line derived from a pheochromocytoma of the rat adrenal medulla; a gift from K.W. Tsim (The Hong Kong University of Science and Technology)] were maintained in DMEM, supplemented with 6% FBS and 6% heat-inactivated horse serum, and incubated at 37°C in a water-saturated 5% CO_2_ incubator. All reagents for cell culture were obtained from Invitrogen (Carlsbad, CA, USA).

*Chemical treatment.* The cells were seeded in 6-well-plates at 500,000 cells/well 24 hr before exposure to dioxin or other chemical treatment for determiniation of AChE activity. TCDD, the most potent congener of dioxins, was purchased from Wellington Laboratories Inc. (Ontario, Canada) and employed at low concentrations of 10^–11^ to 10^–9^ M. We also examined 2,3,7,8-tetrachlorodibenzofuran (TCDF; 10^–8^ M) and 2,3,4,7,8-pentachlorodibenzofuran (PeCDF; 3 × 10^-9^ M) (both from Wellington, Ontario, Canada), forskolin (5 × 10^–5^ M; Sigma, St. Louis, MO, USA), and nerve growth factor (50 ng/mL; Alomone Labs, Jerusalem, Israel). CH223191, an inhibitor of the AhR-dependent pathway (Zhao et al. 2010), was obtained from Sigma and used at a concentration of 10^–6^ M. To examine the role of AhR, we pretreated cells with CH223191 3 hr before incubation with TCDD. The solvent dimethyl sulfoxide (DMSO) was present in all treatments at 0.1%. In the *in vitro* assay, SK-N-SH cell lysate was incubated with TCDD (10^–11^ to 10^–9^ M), BW284c51 (a specific inhibitor of AChE; Sigma), at 2 × 10^–5^ M, or 0.1% DMSO alone (control). After 1 hr incubation at room temperature, enzymatic activity of AChE was determined by the Ellman assay (Ellman et al. 1961). BW284c51 served as an assay control.

*Reporter gene constructs and tranfections.* pAChE-Luc and pAChEm-Luc consist of the human *ACHE* and mouse promoter sequences upstream of a firefly luciferase gene in pGL4.10 and pGL3-Basic vectors (Promega, Madison, WI, USA), respectively. The truncated construct, pAChE-T-Luc, derived from pAChE-Luc, was constructed using sense primer 5´-TTA GAT CTC CTC AGG TGA GTC TC-3´ and antisense primer 5´-TTA AGC TTG GCT GCA GGG CAG-3´. *Bgl*II and *Hin*dIII restriction sites were added at the 5´ ends of sense and antisense primers, respectively, as indicated (underlined). The mutated construct, pAChE-M-Luc, derived from pAChE-Luc, was constructed by site-directed mutagenesis, which we accomplished using mutagenic primers and flanking primers. The mutagenic primers included sense primer, 5´-GTC CGT CTG CGA ATT CTC TGT CTC C-3´, and antisense primer, 5´-GGA GAC AGA GAA TTC GCA GAC GGA C-3´, in which the original sequence of the putative DRE (5´-GCG TG-3´) was replaced by an *Eco*RI restriction site (5´-GAA TTC-3´) as indicated (underlined). The flanking primers included sense primer, 5´-TTA GAT CTA GAT CTC GAG CTC GAG GAT CCC-3´, and antisense primer, 5´-TTA AGC TTC GCC TGC CCT GCA GCC AAG CTT-3´, where *Bgl*II and *Hin*dIII restriction sites, respectively, were added at the 5´ ends of primers as indicated (underlined). PCR (polymerase chain reaction) was performed using Pfx polymerase (Invitrogen). A fragment consisting of the promoter sequences from –1 to –1568 and a fragment with mutation on the 5´ putative DRE were obtained by PCR, and the product was subcloned into the same vector as the full-length pAChE (pGL4.10) via *Bgl*II and *Hin*dIII restriction sites to produce the truncated construct pAChE-T-Luc and the mutated construct pAChE-M-Luc.

Cultured cells were seeded in 24-well plates at 50,000 cells/well 24 hr before being transfected transiently with purified plasmids (0.5 μg/well) and PolyJet™ reagent (SignaGen Laboratories, Rockville, MD, USA) according to the manufacturer’s instructions. The transfection efficiency was approximately 15%.

*Determination of AChE enzymatic activity.* We determined AChE enzymatic activity according to the method of Ellman et al. (1961), modified by the addition of 0.1 mM tetraisopropylpyrophosphoramide (iso-OMPA), an inhibitor of butyrylcholinesterase (BChE). Cells were collected, and total protein extraction was performed at 20°C for 15 min in 200 µL of low-salt lysis buffer (80 mM disodium hydrogen phosphate, pH 7.4) supplemented with 0.5% Triton X-100 and 2.5 mM benzamidine, a protease inhibitor. About 30 µL of cell lysate was incubated with 0.1 mM iso-OMPA and 0.5 mM 5,5´-dithiobis(2-nitrobenzenoic acid) (DTNB) for 30 min at 20°C to inhibit the BChE activity and allow saturation of unspecific reaction with DTNB. This was followed by adding 0.625 mM acetylthiocholine iodide to start the AChE-specific reaction. Absorbance at 410 nm was recorded with a multifunctional microplate spectrometer (TECAN Infinite F200 Pro; Männedorf, Switzerland). Optical density (OD) was recorded at 5-min intervals over a period of 30 min, at 20°C. In this period of time, OD derived from the cell lysate increased linearly with time. The velocity of the reaction was calculated from the slope of the line obtained. Arbitrary units of enzymatic activity are expressed as velocity (mOD per minute) per microgram of protein. All reagents were obtained from Sigma. We measured protein concentrations using a kit from Bio-Rad Laboratories (Hercules, CA, USA) and following the Bradford method (Bradford 1976).

*Luciferase assay.* Cells were transfected with promoter–reporter constructs together with cDNA encoding the β-galactosidase gene at 10:1 weight ratio. Twenty-four hours later, cells were treated with chemicals as described above. For luciferase measurement, sample wells were washed twice with phosphate-buffered saline (PBS), followed by the addition of cell lysis buffer (Promega) and shaking of the plates for 10 min at room temperature to allow cell lysis. Insoluble material was removed by centrifugation, and the resulting lysates were transfered to white 96-well microplates for measurement of luciferase activity using a TECAN Infinite F200 Pro luminometer with automatic injection of Promega stabilized luciferase reagent. Luciferase activity in each well was normalized to total protein and to transfection efficiency as determined by β-galactosidase activity.

*Real-time quantitative PCR.* We isolated total RNA (5 µg) from SK-N-SH cultures using TRIzol reagent (Invitrogen); cDNA was prepared using 5 µg of RNA and Moloney Murine Leukemia Virus Reverse Transcriptase (Invitrogen) according to the manufacturer’s instructions. Real-time PCR of AChE T subunit (*AChE_T_*), AChE R subunit (*AChE_R_*), *PRiMA* (proline-rich membrane anchor), and *18S* rRNA transcripts was performed on equal amounts of cDNA using SYBR Green Master mix and Rox reference dye, according to the manufacturer’s instructions (Applied Biosystems, Foster City, CA, USA). The primers (with GenBank accession numbers; GenBank; http://www.ncbi.nlm.nih.gov/genbank/) were as follows: 5´-GGG GTT CCC CAG GTA AGT GAC CT C-3´ and 5´-T TG AGC AGC GAT CCT GCT TGC TGT AG-3´ for human *AChE_T_* transcript (NM_000665), 5´-CTG GGG TGC GGA TCG GTG TAC CCC-3´ and 5´-TCA CAG GTC TGA GCA GCG TTC CTG-3´ for rat *AChE_T_* (Boudreau-Larivière et al. 2000), 5´-CCC CTG GAC CCC TCT CGA AAC-3´ and 5´-TGG GGA GGA AGC GGT TCC AGA AG-3´ for human *AChE_R_* transcript (AY750146; Birikh et al. 2003), 5´-TCT GAC TGT GCT TGT CAT CAT TTG CTA C-3´ and 5´-AGG GCC TGC AGA CTC ACA CCA C-3´ for human *PRiMA* transcript (NM_178013), and 5´-GAC TGT TAT GGT CAA GGT GAA-3´ and 5´-GAT AGT CAA GTT CGA CCG TC-3´ for human *18S* rRNA (NR_003286; Guo et al. 2011). The SYBR green signal was detected by MX3005P multiplex quantitative PCR system (Stratagene, La Jolla, CA, USA). The relative transcript expression levels were quantified using the ΔΔCT method (Winer et al. 1999). The specificity of amplification was confirmed by melting curves and by gel electrophoresis.

*MTT [3-(4,5-dimethylthiazol-2-yl)-2,5-diphenyltetrazolium bromide] assay.* For cell viability tests, cultured SK-N-SH cells in 96-well plates were treated with TCDD for 48 hr, followed by the addition of MTT in PBS at final concentration of 0.5 mg/mL for 2 hr. The medium was aspirated, and the cultures were resuspended in 150 µL DMSO to determine cell viability by absorbance at 570 nm.

*Other assays.* To determine β-galactosidase enzymatic activity, 20 µL of cell lysate was mixed with 80 µL of sodium phosphate buffer (pH 7.5) containing 0.8 mg/mL *o*-nitrophenyl-β-d-galactopyranoside. After incubation at 37°C for 1 hr, absorbance was measured at 410 nm. In this period of time, OD derived from the cell lysate varied linearly with time.

*Statistics.* We performed statistical tests using Origin Pro software (version 8; OriginLab, Northampton, MA, USA). One-way analysis of variance (ANOVA) was used for most analyses, but two-way ANOVA was used for promoter truncation and mutation studies. We used the Bonferroni test to perform means comparisons between two treatment groups. We considered *p* < 0.05 to be statistically significant.

## Results

**Figure 1:**
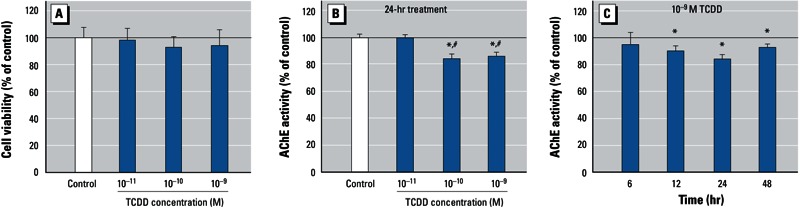
Effects of TCDD on cell viability (*A*) and the enzymatic activity of AChE (*B,C*) in cultured neuronal SK‑N‑SH cells incubated with TCDD (10–11 to 10–9 M) or 0.1% DMSO (control) for 6–48 hr. (*A*) Cell viability was assessed by MTT assay 48 hr after exposure. Dose response (*B*) and time course (*C*) showing suppression of AChE activity by TCDD. See “Materials and Methods” for additional details. Values were calculated as a percentage of control and are expressed as mean ± SE (*n* = 4); each independent sample was tested in triplicate. *p < 0.05 compared with control by one-way ANOVA with Bonferroni test. ^#^*p* < 0.05 compared with 10–11 M TCDD by one-way ANOVA with Bonferroni test.

*Dioxin decreases the enzymatic activity of neuronal AChE.* We investigated the effects of dioxin on neuronal AChE activity by exposing quiescent human SK-N-SH neroblastoma cells to TCDD (10^–11^ to 10^–9^ M) for 48 hr. MTT assays revealed no obvious cell death (Figure 1A). We also examined the effects of treatment, time, and TCDD concentration on the enzymatic activity of AChE. The enzymatic activity of AChE was reduced by approximately 15% after 24 hr exposure to 10^–10^ M and 10^–9^ M TCDD compared with DMSO-treated controls (mean ± SE, 2.8 ± 0.18 mOD/min/μg) (Figure 1B). The lowest concentration of TCDD (10^–11^ M) had no effect on AChE activity (Figure 1B). Because the 10^–10^ M and 10^–9^ M groups were not significantly different, we used 10^–9^ M TCDD dose for the time-course experiments. We observed significant decreases in AChE activity after 12, 24, and 48 hr of exposure to 10^–9^ M TCDD compared with controls, but there were no significant differences between effects at the various time points. However, in the same time-course experiments, AChE activity in all the control groups varied with time. In controls, the mean values of the 6- to 24-hr groups varied little (2.8–3.1 mOD/min/μg) but that of 48-hr group was relatively high (5.7 ± 0.08 mOD/min/μg). The relatively high AChE activity in the 48-hr group might be a result of the higher cell density of the cultures at harvest. Moreover, we observed decreased AChE activity in SK-N-SH cells exposed to two other dioxins: TCDF (~ 74% of control) and PeCDF (~ 68% of control) [see Supplemental Material, Table S1 (http://dx.doi.org/10.1289/ehp.1206066)].

**Figure 2:**
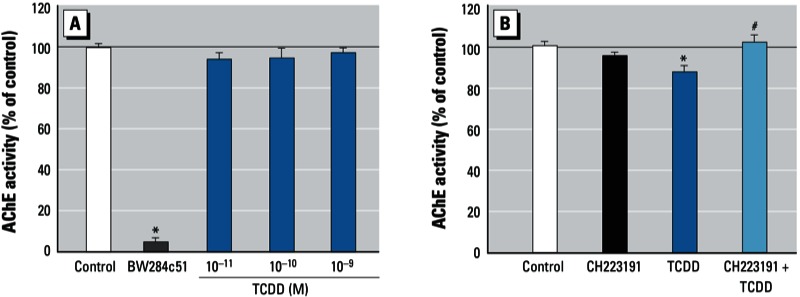
Effects of TCDD on AChE activity via direct inhibition of the enzyme (*A*) and via the AhR-dependent pathway (*B*). (*A*) AChE activity in cell lysate of SK‑N‑SH incubated with TCDD (10^–11^ to 10^–9^ M), 2 × 10^–5^ M BW284c51 (AChE inhibitor used as the positive control), or with 0.1% DMSO (control) for 1 hr. (*B*) AChE activity in cultured SK‑N‑SH cells incubated with 10^–6^ M CH223191 (AhR inhibitor) or 0.1% DMSO for 3 hr and then incubated with 10^–9^ M TCDD or 0.01% DMSO for 24 hr. See “Materials and Methods” for additional details. Values were calculated as a percentage of control and are expressed as mean ± SE (*n* = 4); each independent sample was tested in triplicate. *p < 0.05 compared with control by one-way ANOVA with Bonferroni test. ^#^*p* < 0.05 compared with TCDD alone by one-way ANOVA with Bonferroni test.

*Dioxin does not directly inhibit AChE enzyme.* After determining that dioxin decreased AChE activity, we next examined whether dioxin could directly inhibit AChE enzymatic activity in an *in vitro* assa*y.* TCDD was mixed with cell lysate of SK-N-SH cultures for 1 hr at 20°C. None of the TCDD concentrations tested (10^–11^ to 10^–9^ M) inhibited the activity, whereas addition of BW284c51 (2 × 10^–5^ M), a specific AChE inhibitor, to the cell lysate inhibited nearly 90% of the enzymatic activity (Figure 2A). These results suggest that dioxin is unlikely to act by direct inhibition of AChE and that the suppressive effects of dioxin on AChE activity may occur via transcriptional regulation.

*Dioxin suppresses AChE activity via AhR.* When AhR is activated by dioxins, it translocates into the nucleus and forms a heterodimer with its partner ARNT (aryl hydrocarbon receptor nuclear translocator). The heterodimer binds to the DRE in the promoter region upstream of target genes and thus regulates transcription. The role of AhR in the dioxin-induced decrease of AChE activity was first investigated using CH223191, a ligand-selective antagonist of the AhR, which can preferentially inhibit effects of certain classes of AhR agonists, including TCDD (Zhao et al. 2010). We found that, compared with DMSO (mean ± SE, 3.8 ± 0.18 mOD/min/μg), 10^–9^ M TCDD significantly decreased the activity (Figure 2B), consistent with the result in Figure 1B. In contrast, pretreatment with CH223191 blocked this decrease, indicating that AhR was involved in the dioxin-induced effect.

**Figure 3:**
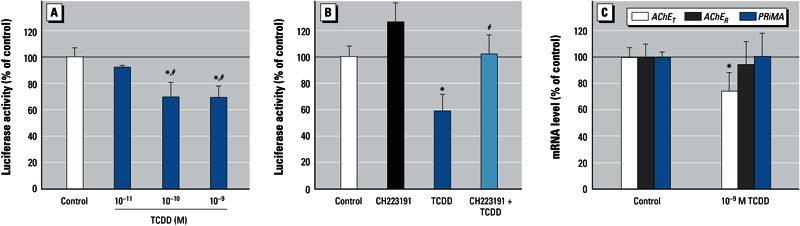
Effect of TCDD on promoter activity (*A,B*) and mRNA levels (*C*) in SK‑N‑SH cells. (*A, B*) The human ACHE promoter–reporter construct (pAChE-Luc) was transiently transfected into SK‑N‑SH cells 1 day before treatment; after 24 hr of treatment, promoter activity was determined by luciferase assays. (*A*) Transfected cells were incubated with TCDD (10^–11^ to 10^–9^ M) or with 0.1% DMSO (control). (*B*) Transfected cells were pretreated with 10^–6^ M CH223191 (AhR inhibitor) for 3 hr and then incubated for 24 hr with 10^–9^ M TCDD or 0.01% DMSO. (*C*) Expression level of AChE transcripts (AChET and AChER variant) and PRiMA transcripts determined by real-time PCR analysis of total RNA extracted from cells treated with 10^–9^ M TCDD or 0.1% DMSO. See “Materials and Methods” for additional details. Values were calculated as a percentage of control and are expressed as mean ± SE (*n* = 3); each independent sample was tested in triplicate. *p < 0.05 compared with control by one-way ANOVA with Bonferroni test. ^#^*p* < 0.05 compared with TCDD by one-way ANOVA with Bonferroni test.

*Dioxin causes transcriptional down-regulation of AChE.* Using a human *ACHE* promoter–driven luciferase reporter construct (pAChE-Luc) with approximately 2.2 kb of the regulatory region upstream of the human *ACHE* gene, we evaluated TCDD’s effects on the promoter activity of human AChE. This construct has been well characterized and extensively used to study the regulation of the *ACHE* gene (Getman et al. 1995; Siow et al. 2002). Quiescent SK-N-SH cells were transiently transfected with pAChE-Luc 1 day before the application of TCDD (10^–11^ to 10^–9^ M). Promoter activity was determined by luciferase assay after 24 hr of TCDD treatment. Consistent with AChE activity, we observed a significant decrease (~ 30%) in human *ACHE* promoter activity after TCDD exposure (10^–10^ to 10^–9^ M) compared with the DMSO control (Figure 3A). Similar to the effects on AChE activity, pretreatment with CH223191 (10^–6^ M) significantly reversed the suppressive effect of TCDD (10^–9^ M) on the promoter activity of human *AChE*, consistent with our assumption that an AhR-dependent pathway directs dioxin-induced transcriptional suppression of AChE (Figure 3B).

The transcriptional regulation of AChE by dioxin was further confirmed by real-time PCR analyses to determine expression levels of *AChE_T_* mRNA (the major AChE transcript in neurons), *AChE_R_* mRNA (the minor AChE transcript in the brain), and *PRiMA* mRNA (a structural subunit of the active form of neuronal AChE) (reviewed by Massoulié 2002). Results showed an approximately 25% decrease in *AChE_T_* mRNA in response to 10^–9^ M TCDD, with no significant changes in *AChE_R_* and *PRiMA* mRNA levels (Figure 3C). Similar to the change in *AChE* mRNA, the protein level of AChE was obviously reduced after TCDD exposure [see Supplemental Material, Figure S1 (http://dx.doi.org/10.1289/ehp.1206066)]. Therefore, we conclude that exposure to dioxin leads to a decrease in the mRNA expression of AChE catalytic subunit, resulting in decreased expression of the active form of AChE.

**Figure 4:**
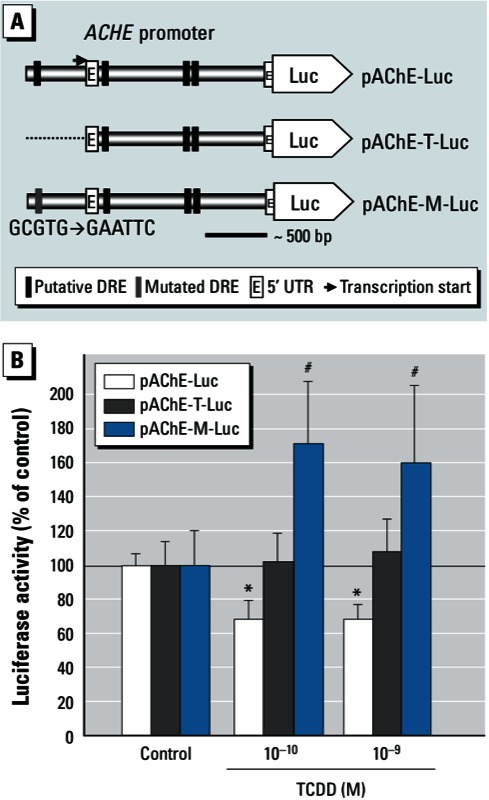
Putative DREs in the human AChE promoter and their response to TCDD exposure. (*A*) Putative DRE consensus sequences in the pAChE-Luc construct (full-length; top), pAChE-T-Luc (truncated; middle), and pAChE-M-Luc (mutant; bottom). (*B*) The three constructs were transiently transfected into cultured SK‑N‑SH cells 1 day before 24 hr incubation with TCDD (10^–10^ to 10^–9^ M) or 0.1% DMSO (control); luciferase assays were then performed to determine the promoter activity. See “Materials and Methods” for additional details. Values were calculated as a percentage of control and are expressed as mean ± SE (*n* = 3); each independent sample was tested in triplicate. *p < 0.05 compared with control by one-way ANOVA with Bonferroni test. ^#^*p* < 0.05 compared with pAChE-Luc transfected cells by two-way ANOVA with Bonferroni test.

*Putative DRE(s) in the human AChE promoter.* The presence of DREs in the regulatory region upstream of dioxin-responsive genes is a key component of the AhR-dependent signaling pathway in response to dioxin. We found four putative consensus core sequences of DRE (5´-TNGCGTG-3´ or 5´-CACGCNA-3´) (Nukaya et al. 2009) within the approximately 2.2-kb region upstream of the human *ACHE* gene (GenBank NM_000665). All of the putative DREs are exact matches or reverse complements of 5´-GCGTG-3´ [see Supplemental Material, Figure S2 (http://dx.doi.org/10.1289/ehp.1206066)]. Considering that the 5´ DRE is the only one located upstream of the transcription start sites among all four putative DREs (Getman et al. 1995; Figure 4A), we concentrated our investigation on the role of this putative DRE. In cultures transfected with truncated human AChE promoter without the 5´ DRE site (pAChE-T) or with a promoter containing a specific mutation of the DRE site (pAChE-M), we found that TCDD no longer suppressed promoter activity (Figure 4B). Furthermore, the effect of 10^–10^ M and 10^–9^ M TCDD on pAChE-M was significantly different from that on the wild type (pAChE). These results suggest that the 5´ DRE may play a critical role in mediating suppression.

**Figure 5:**
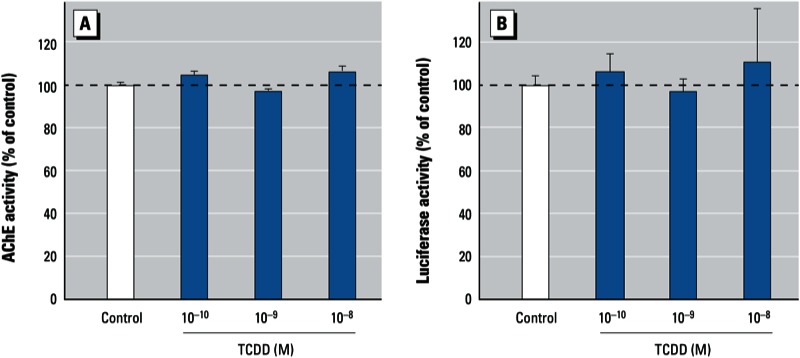
Effect of TCDD (10^–10^ to 10^–8^ M) on AChE enzymatic activity (*A*) and promoter activity (*B*) in PC12 rat neuronal cells. (*A*) AChE enzymatic activity in cells incubated with TCDD or 0.1% DMSO (control) for 24 hr. (*B*) The mouse ACHE promoter–reporter construct (pAChEm-Luc) was transiently transfected into PC12 cells 1 day before treatment; after 24 hr of treatment with TCDD or DMSO, promoter activity was determined by luciferase assays. Values were calculated as a percentage of control and are expressed as mean ± SE (*n* = 3); each independent sample was tested in triplicate.

*Differential responses of rodent AChE to dioxin.* Because rodent AChE genes lack obvious DREs [Sun et al. 2004; see Supplemental Material, Figure S2 (http://dx.doi.org/10.1289/ehp.1206066)], we tested the effects of dioxin on AChE in PC12 cells, a rat neuronal cell line widely used in toxicology studies. After 24 hr exposure, none of the TCDD treatment groups (10^–10^, 10^–9^, or 10^–8^ M) showed suppression of the AChE activity (Figure 5A). In a similar manner, TCDD exposure had no effect on promoter activity of mouse *ACHE* when we used a construct (pAChEm-Luc) consisting of an approximately 2.1-kb regulatory region upstream of the mouse *ACHE* gene driving luciferase reporter gene expression (Figure 5B) (Jiang et al. 2003). The absence of consensus DRE sequences in the 2.1-kb regulatory region upstream of the mouse *ACHE* gene may help to explain the different responses of rodent and human *ACHE* genes (Sun et al. 2004). PC12 cells exhibited normal responses to treatment with forskolin, which activates the cAMP-dependent pathway, and nerve growth factor. We observed neurite outgrowth and an increment in AChE activity in cells treated with forskolin and nerve growth factor, respectively (see Supplemental Material, Figure S3). These data further support the explanation that the unresponsiveness to TCDD may be due to the absence of DRE on the promoter of rodent *ACHE*.

## Discussion

Emerging evidence has shown effects of dioxin in the central cholinergic system. Ahmed (2011) reported that daily administration of TCDD (0.2 or 0.4 µg/kg body weight) to pregnant rats from gestation day 1 to lactation day 30 interfered with the development of AChE expression in cerebella of the offspring, although there was no obvious sign of developmental toxicity. Ahmed (2011) suggested that the effects of TCDD on brain AChE were related to alterations in thyroid development. Ahmed’s *in vivo* study suggested that TCDD is able to affect AChE in the brain. In the present *in vitro* study, we observed that TCDD suppressed AChE activity in cultured human neuronal cells. However, AChE may not be the only target of dioxin in the cholinergic system, because putative DRE sites are also present in the promoter region of acetylcholine receptor (Sun et al. 2004). Thus, our finding on AChE is a starting point for the exploration for other abnormalities in cholinergic function directly caused by dioxin.

AChE may have functions besides the classical function in cholinergic neurotransmission; evidence has suggested functions in, for example, synapse transmission (reviewed by Zimmerman and Soreq 2006), neurite outgrowth (reviewed by Paraoanu and Layer 2008), apoptosis (reviewed by Jiang and Zhang 2008), and bone formation (Vogel-Hopker et al. 2012). Thus, the interference of dioxin with AChE might bring new insight into the biological or toxicological effects mediated by AhR not only in the nervous system but also elsewhere in the human body.

Monitoring of AChE inhibition has been used as an indicator of OP exposure (reviewed by Chen et al. 1999; Farahat et al. 2011). Emerging evidence suggests that other types of xenobiotics, such as heavy metals (Richetti et al. 2011) and nanoparticles (Wang et al. 2009), can also affect AChE activity. Here we provide evidence of neuronal AChE regulation by another type of xenobiotic, dioxins. Our results show that AhR mediated the transcriptional regulation of AChE. Considering the ability of AhR to bind diverse structures of chemicals (Zhao et al. 2010), the spectrum of xenobiotics affecting AChE transcription could extend beyond dioxin-like chemicals. These results suggest that in the future, in addition to assaying for AChE activity, it may be possible to monitor exposures by demonstrating down-regulation of AChE transcripts.

In our experiments, the alterations in neuronal AChE expression were induced by low concentrations of TCDD, close to environmental levels. In several accidental exposures to dioxins, such as in Vietnam (Tai et al. 2011), Seveso, Italy (Needham et al. 1997), and Taiwan (Guo et al. 2004), exposed individuals had median serum levels of approximately 1,000, 450, and 180 pg/g fat, respectively. Based on the estimated average serum fat content of 6.9 g/L (Phillips et al. 1989), the average serum concentration of dioxin (TCDD) in these individuals would be 10^–10^ to 10^–11^ M. Although concentrations of dioxins in brain tissue of exposed individuals are unknown, we based our experimental concentrations on these serum concentrations and on concentrations used in other studies (Jin et al. 2004; Sánchez-Martín et al. 2010). In the present study using relatively low concentrations of TCDD, cultured neuronal cells exhibited no significant change in viability, which made it feasible to study the functional alterations induced by dioxin.

On the basis of the present findings and the literature (reviewed by Beischlag et al. 2008), we assume that

When dioxin enters the neuronal cells, it will bind to AhR in the cytosol, resulting in the transformation and translocation of the receptor.
The active AhR then goes into the nucleus and binds with ARNT to form a heterodimer.
The heterodimer will then bind to the putative DRE site(s) on the promoter region of the ACHE gene, suppressing the expression of AChET transcripts.
This transcriptional suppression of the major neuronal AChE transcript leads to a decrease in the production of the AChE catalytic subunits and finally causes the decrease in enzymatic activity.

However, further investigations are needed to clarify the role of the putative DRE(s) and how transcriptional suppression occurs. Apart from this transcriptional mechanism, we found no evidence showing that dioxin could inhibit AChE activity by direct interaction with the catalytic subunit.

Toxic effects of environmental chemicals can be species specific. For example, the binding affinity of dioxin to AhR is higher in mice than in humans (reviewed by Denison et al. 2011), and endocrine-disrupting chemicals, such as bisphenol A and its analogs, are potent agonists for human pregnane X receptor (hPXR) but do not affect mouse PXR activity (Sui et al. 2012). Our findings show that dioxin has a suppressive effect on AChE expression in human, but not rat, neuronal cells (Figure 5). The species specificity found in the present study is unlikely to have been caused by differences in affinities of dioxin–AhR binding, but rather resulted from the presence and absence of DREs in the regulatory regions of human and rodent *ACHE* genes, respectively. This finding highlights the advantage of *in vitro* toxicity testing using human cell lines instead of animal-derived cell lines in assessment of effects of human exposure to xenobiotics, as proposed by Tox21 (Bhattacharya et al. 2011; Hartung 2009; reviewed by Krewski et al. 2010).

## Conclusion

We found a novel mechanism whereby dioxin may produce its biological or toxicological effects by decreasing neuronal AChE activity through a transcriptional down-regulation mechanism via the AhR-dependent pathway. To our knowledge, this is the first study to report direct interference by dioxin with the cholinergic neurotransmission system.

## Supplemental Material

(688 KB) PDF
