# Supplementary material for: AhR-Mediated Effects of Dioxin on Neuronal Acetylcholinesterase Expression in Vitro
Source: Environ Health Perspect. 2013 Feb 20;121(5):613–8. doi: 10.1289/ehp.1206066 (PMC3673198; doi:10.1289/ehp.1206066)
Supplement: (688 KB) PDF [file ehp.1206066.s001.pdf]

Supplemental Material

AhR-Mediated Effects of Dioxin on Neuronal Acetylcholinesterase Expression in Vitro

Heidi Qunhui Xie, Hai-Ming Xu, Hua-Ling Fu, Qin Hu, Wen-Jing Tian, Xin-Hui Pei and Bin Zhao

Table of Contents

Supplemental Material, Figure S1	3
Supplemental Material, Figure S2	4
Supplemental Material, Figure S3	5
Supplemental Material, Table S1	6

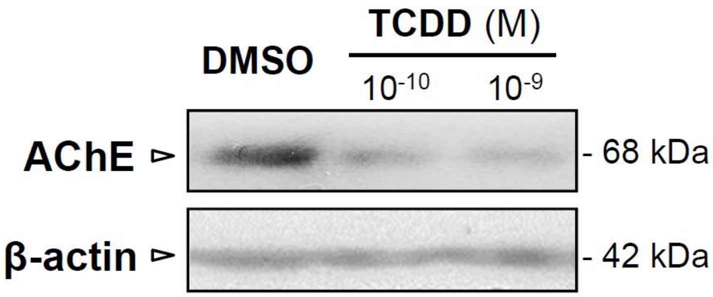

Supplemental Material, Figure S1: Change in AChE protein upon TCDD treatment. The protein level of AChE was determined by Western blot assay. Cultures of SK-N-SH were treated with TCDD (10^{-10} ~10^{-9} M) or DMSO (0.1%) for 24 hours. After the treatment, the cultures were collected immediately in lysis buffer containing 125 mM Tris-HCl (pH 6.8), 2% SDS, 10% glycerol and 200 mM 2-mercaptoethanol, and the proteins were subjected to 8% SDS-PAGE electrophoresis. After transfer, the membrane was incubated with anti-AChE antibody (1: 1000; Santa Cruz, Santa Cruz, CA) and anti-β actin antibody (1: 10000; Sigma) at 4 ^{o}C for 16 hours for the protein detection. The immuno-complexes were visualized by the enhanced chemiluminescence (ECL) method (Millipore, Billerica, MA). An obvious reduction of AChE protein was observed, *n*=3.

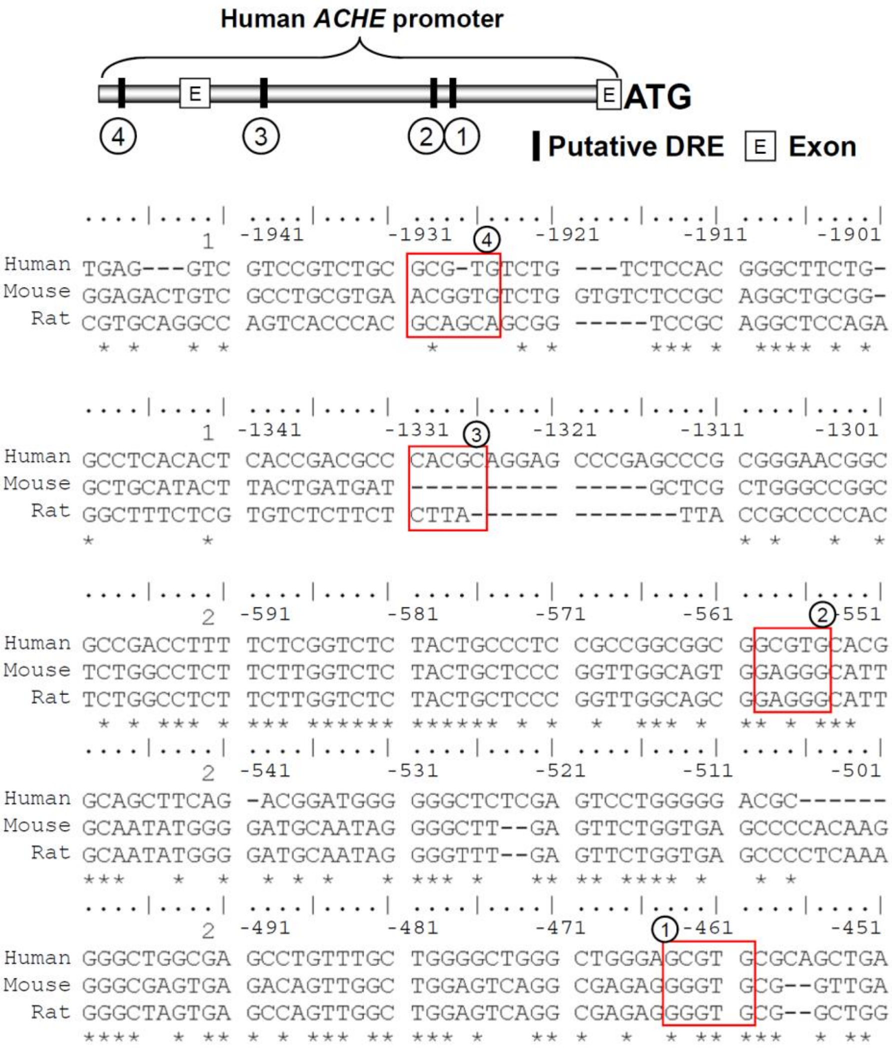

Supplemental Material, Figure S2: Sequence of the putative DRE sites on the promoter region of human (NC_000007) *ACHE* and the alignments with the corresponding mouse (NC_000071) and rat (NC_005111) *ACHE* promoter sequences are shown. The promoter sequences are distinct among different species and an absence of the DRE consensus sequence is observed in mouse and rat.

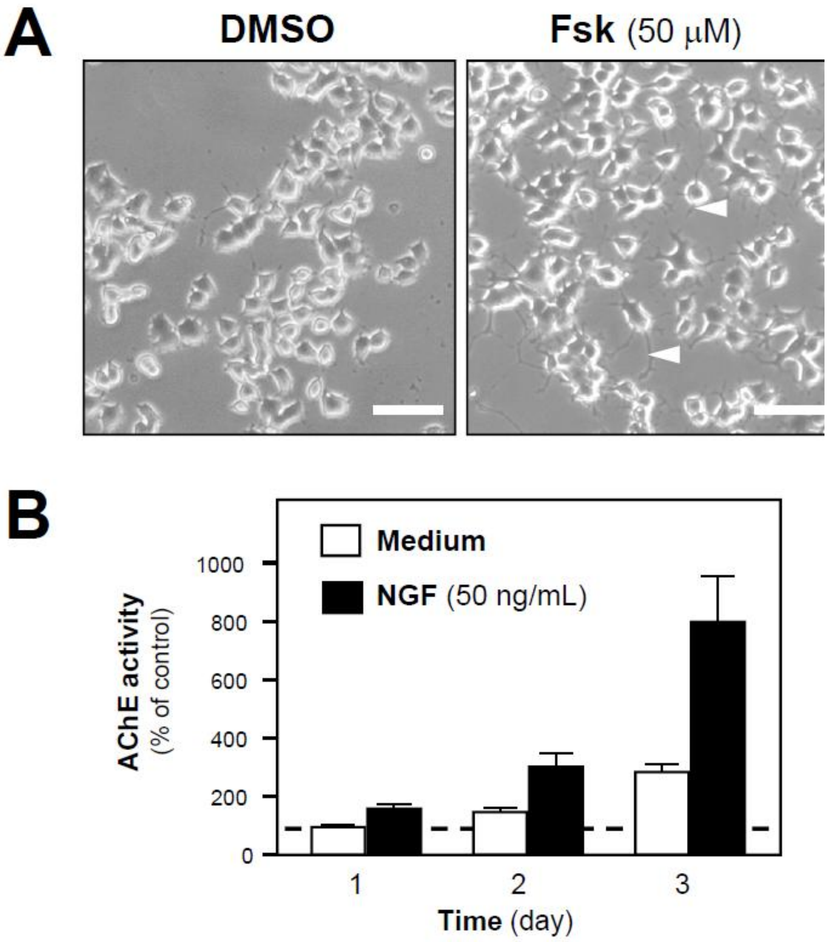

Supplemental Material, Figure S3: Responsiveness of the cultured PC12 cells to chemicals. (*A*) Cultured PC12 cells were treated with forskolin (Fsk) at 50 μM for 72 hours. The treated cells were fixed with ice-cold 4% paraformaldehyde and the extension of neurites was observed as pointed by arrows. Bar = 50 μm. (*B*) Cultured PC12 cells were treated with nerve growth factor (NGF) at 50 ng/mL. Cell lysates were collected at indicated time points to determine the activity of AChE by Ellman assay. An obvious increase in AChE activity was observed after NGF treatment.

Dioxins	TEF(WHO)	Concentration	AChE activity (% of Control)	*p* Value (Compared to control)
2,3,7,8-TCDF	0.1	10^{-8} M	73.8±6.1	0.02
2,3,4,7,8-PeCDF	0.3	$3x10^{-9}$ M	67.8±2.6	0.009

Supplemental Material, Table S1: Effects of other dioxins on AChE activity in SK-N-SH cells. Cultured SK-N-SH cells were treated with 2,3,7,8-tetrachlorodibenzofuran (TCDF) (Wellington, Ontario, Canada) at 10^{-8} M or 2,3,4,7,8-pentachloro- dibenzofuran (PeCDF) (Wellington, Ontario, Canada) at $3x10^{-9}$ M or solvent alone (Control) at 0.01% for 24 hours. Enzymatic activity of AChE was determined as described in Materials and Methods. Values are calculated as % of solvent alone (Control) and expressed as mean±SEM, *n*=3, each independent sample was tested in triplicate. Bonferroni test was used for means comparisons between solvent control and dioxin treatment; *p* values were obtained by one-way ANOVA with Bonferroni test compared with solvent-treated cells.
